# Photobiomodulation improves acute restraint stress-induced visceral hyperalgesia in rats

**DOI:** 10.1007/s10103-024-04091-2

**Published:** 2024-05-28

**Authors:** Naoya Ishibashi, Takuya Nanjo, Shinichi Tao

**Affiliations:** Bio-medical Engineering Group, Drug Discovery Laboratory, Teijin Institute for Bio-medical Research, Teijin Pharma Ltd., 4-3-2, Asahigaoka, Hino-shi, Tokyo 191-8512 Japan

**Keywords:** Photobiomodulation, Low level laser therapy, Irritable bowel syndrome, Visceral hyperalgesia

## Abstract

The purpose of this study is to explore the potential application of photobiomodulation to irritable bowel syndrome. We established the following experimental groups: the Non-Stress + Sham group, which consisted of rats that were not restrained and were only subjected to sham irradiation; the Stress + Sham group, which underwent 1 hour of restraint stress followed by sham irradiation; and the Stress + Laser group, which was subjected to restraint stress and percutaneous laser irradiation bilaterally on the L6 dorsal root ganglia for 5 minutes each. The experiment was conducted twice, with three and two laser conditions examined. Following laser irradiation, a barostat catheter was inserted into the rat’s colon. After a 30-minute acclimatization period, the catheter was inflated to a pressure of 60 mmHg, and the number of abdominal muscle contractions was measured over a 5-minute period. The results showed that photobiomodulation significantly suppressed the number of abdominal muscle contractions at average powers of 460, 70, and 18 mW. However, no significant suppression was observed at average powers of 1 W and 3.5 mW. This study suggests that photobiomodulation can alleviate visceral hyperalgesia induced by restraint stress, indicating its potential applicability to irritable bowel syndrome.

## Introduction

Irritable bowel syndrome (IBS) is a chronic gastrointestinal disorder characterized by recurrent abdominal pain and associated with abnormal stool form or frequency [1]. Typically, clinical and endoscopic examinations fail to identify the organic cause of these symptoms, leading to IBS being classified as a functional bowel disorder [2]. In recent years, it has been grouped with similar disorders such as functional dyspepsia and fibromyalgia, collectively referred to as disorders of gut-brain interactions (DGBI) [3]. The prevalence of IBS varies significantly depending on the diagnostic criteria used, but it is estimated that it affects 5 to 20% of the general population [4]. Common symptoms reported by many IBS patients include abdominal pain, bloating, and abnormal bowel movements (diarrhea, constipation) [1], and it has been reported that their quality of life is significantly impaired [5]. There are also reports of decreased work productivity [6], which is suggested to be potentially higher compared to diseases like diabetes [7].

The pathophysiological mechanism of IBS remains unclear, and several mechanistic hypotheses have been proposed [8]: abnormalities in the intestinal bacterial layer [9], the hormone corticotropin-releasing hormone produced by psychosocial stress [10], inflammation of the intestinal mucosa [11], increased mucosal permeability [12], central sensitization [13], and genetic factors [14]. Various treatments have been proposed based on these hypotheses, but so far, no single treatment has proven effective in alleviating symptoms.

We propose the potential application of photobiomodulation (PBM) to IBS. PBM is a treatment method that utilizes the biological effects of light from sources such as lasers and light emitting diodes [15], and has been shown to have an analgesic effect [16–18]. It is believed to inhibit the activity of Aδ and C fibers that transmit pain [19–22]. Because visceral pain from rectal hypersensitivity is also transmitted through Aδ and C fibers [23, 24], PBM may be effective for colonic hypersensitivity, a typical symptom of IBS.

In this study, we used an acute restraint stress model, one of the IBS models, to explore the potential application of PBM to IBS.

## Materials and methods

This study was conducted with the approval of the Animal Experiment Committee of Nihon Bioresearch Inc. (approval No. 390216 and 409079) and Teijin Pharma Ltd. (approval No. B19-040-R and B20-007).

### Construction and evaluation of restraint stress model

We utilized male rats (Crlj: WI, 6 weeks old) for this study. Each rat was individually housed in stainless steel hanging cages (24 × 38 × 20 cm). Solid feed (CRF-1, Oriental Yeast Co., Ltd.), manufactured within the past 9 months, was placed in the feeder for free ingestion. The animals were quarantined for 5 days and then acclimated for 6 days. 3 days prior to the restraint stress test, the dorsal part of the target animal was depilated. Specifically, the lumbar sacral area was widely depilated using an electric clipper and depilatory cream (Epilat depilatory cream, Kracie). The animals were kept in a breeding room maintained at a temperature of 20.0–26.0 °C (actual value: 22.8–23.4 °C), humidity of 40.0–70.0% (actual value: 48.8–53.7%), with a 12-hour light-dark cycle (lighting: 6:00–18:00), and with a ventilation rate of 12 times/hour (fresh air through a filter).

The experiments were conducted during the light cycle. Rats in the Stress + Sham, which underwent one hour of restraint stress followed by sham irradiation, and Stress + Laser groups, which was subjected to restraint stress and percutaneous laser irradiation, were placed in a restraint stress cage (4.5 × 4.5 × 18.0 cm) for 1 hour to induce restraint stress. Rats in the Non-Stress + Sham group, which consisted of rats that were not restrained and were only subjected to sham irradiation, were kept in their home cages. Subsequently, in the Stress + Laser group, one individual restrained the rat by hand, while another applied a laser irradiation probe to the shaved skin of the rat’s lumbar region, delivering percutaneous laser irradiation to the L6 dorsal root ganglion for 5 minutes on each side. The L6 dorsal root ganglion was chosen as the site for laser irradiation because it is said to receive projections from the nerves originating from the rectum [25]. The Non-Stress + Sham and Stress + Sham groups underwent similar procedures without laser irradiation. Thereafter, a barostat catheter was inserted into the colon of rats in all groups, and the rats were acclimatized to the catheter for 30 minutes. After acclimatization, the catheter was inflated to a pressure of 60 mmHg, and the number of abdominal contractions was counted over a period of 5 minutes. The evaluator conducted the assessments blind to the procedures the rats had undergone. Each experimental group consisted of 10 animals for the Non-Stress + Sham group and 15 animals for the Stress + Sham and Stress + Laser groups, respectively.

### Laser irradiation conditions for restraint stress model

The test was conducted twice, with three laser intensity conditions in the first test and two laser intensity conditions in the second test. The laser conditions for each test are shown in Tables 1 and 2. A semiconductor laser source (ML6500 system; Modulight Corporation, Tampere, Finland) was used. The laser light was guided by an optical fiber from the laser source. Laser power, irradiation time, and oscillation mode were controlled using laser source software (ML6700 Controller; Modulight Corporation, Tampere, Finland). Average powers of 70, 18, and 3.5 mW were achieved by placing a neutral density filter (AND-10 C series; SIGMAKOKI Company, Limited, Tokyo, Japan) behind the optical fiber, and 1000 and 460 mW were emitted directly from the optical fiber without using the neutral density filter. Average power was measured using a power meter (display; NOVAII, sensor; 10 A-1.1 V; Ophir Japan Limited, Saitama, Japan).

**Table 1:** Laser parameters for first experiment (Fig. 2)

Parameters	Stress + Laser1	Stress + Laser2	Stress + Laser3
Wavelength	808 nm		
Average power	1000 mW	70 mW	18 mW
Peak power	10 W	700 mW	180 mW
Area	2.79 cm^2^		
Average power density	358 mW/cm^2^	25.1 mW/cm^2^	6.45 mW/cm^2^
Irradiation time	300 s per each side (total: 600 s)		
Energy	600 J	42 J	10.8 J
Energy density	215 J/cm^2^	15.1 J/cm^2^	3.87 J/cm^2^
Mode	Pulse		
Pulse width	20 ms		
Frequency	5 Hz		
Pulse duty	10%		

**Table 2:** Laser parameters for second experiment (Fig. 3)

Parameters	Stress + Laser4	Stress + Laser5
Wavelength	808 nm	
Average power	460 mW	3.5 mW
Peak power	4.6 W	35 mW
Area	2.79 cm^2^	
Average power density	165 mW/cm^2^	1.25 mW/cm^2^
Irradiation time	300 s per each side (total: 600 s)	
Energy	276 J	2.1 J
Energy density	98.9 J/cm^2^	0.75 J/cm^2^
Mode	Pulse	
Pulse width	20 ms	
Frequency	5 Hz	
Pulse duty	10%	

### Immunohistochemistry

After measuring the number of abdominal contractions, the animals were perfused with saline and 4% paraformaldehyde (PFA) under isoflurane anesthesia 2 hours ± 10 minutes after laser irradiation. The spinal cord, along with the vertebrae, was harvested, immersed in 4% PFA, and stored at 4 °C. The L6 spinal cord was embedded in paraffin blocks and stained for selected marker proteins using the 3,3’-diaminobenzidine (DAB) staining. The marker proteins chosen were c-Fos, phosphorylated ERK1/2 (p-ERK1/2), metabotropic glutamate receptor 5 (mGluR5), and transient receptor potential vanilloid 1 (TRPV1). The following antibodies were used: Anti-c-Fos antibody (ab208942, Abcam), p44/42 MAPK (Erk1/2) (137F5, #4695, Cell Signaling Technology), Anti-Metabotropic Glutamate Receptor 5 antibody (ab76316, Abcam), and Rat Vanilloid R1/TRPV1 Affinity Purified Polyclonal Ab (AF3066, R&D Systems).

### Data analysis

Data are expressed as mean ± standard error of the mean. Statistical tests were conducted to determine whether the disease was induced through a two-group comparison test between the Non-stress + Sham group and the Stress + Sham group, and a multiple comparison test between the Stress + Sham group and multiple Stress + Laser groups to determine whether the laser was effective. The two-group comparison test confirmed the homogeneity of variance by the F-test of variance ratio, and since the variance was confirmed to be equal, Student’s t-test was performed. The multiple comparison test was conducted by performing Bartlett’s test for homogeneity of variance, and since the variance was confirmed to be equal, Dunnett’s test was performed. The significance level was set at 5%, and it was divided into less than 5% (*p* < 0.05) and less than 1% (*p* < 0.01). The commercially available statistical program (SAS system, SAS Institute Japan Co., Ltd.) was used for the significance test.

## Result

**Fig. 1:**
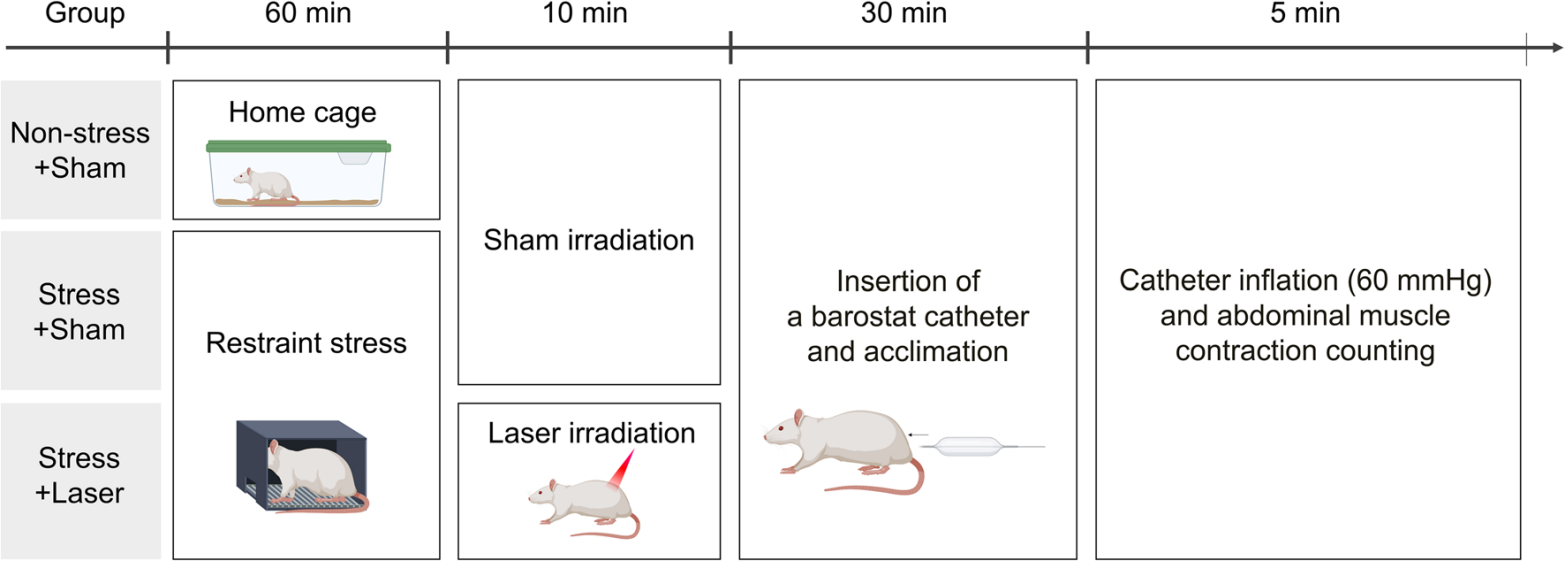
This figure outlines the protocol where Stress + Sham and Stress + Laser groups were subjected to 1 hour of restraint stress, while the Non-Stress + Sham group was kept in home cages. Following this, only the Stress + Laser group received percutaneous laser irradiation on the lumbar region for 5 minutes on each side targeting the L6 dorsal root ganglion, with the other groups undergoing sham procedures. All groups had a barostat catheter inserted into the colon and were allowed 30 minutes for acclimatization before the catheter was inflated to 60 mmHg to measure abdominal contractions over 5 minutes, with all assessments conducted by an evaluator blinded to group assignments. This figure is created with Biorender.com

**Fig. 2:**
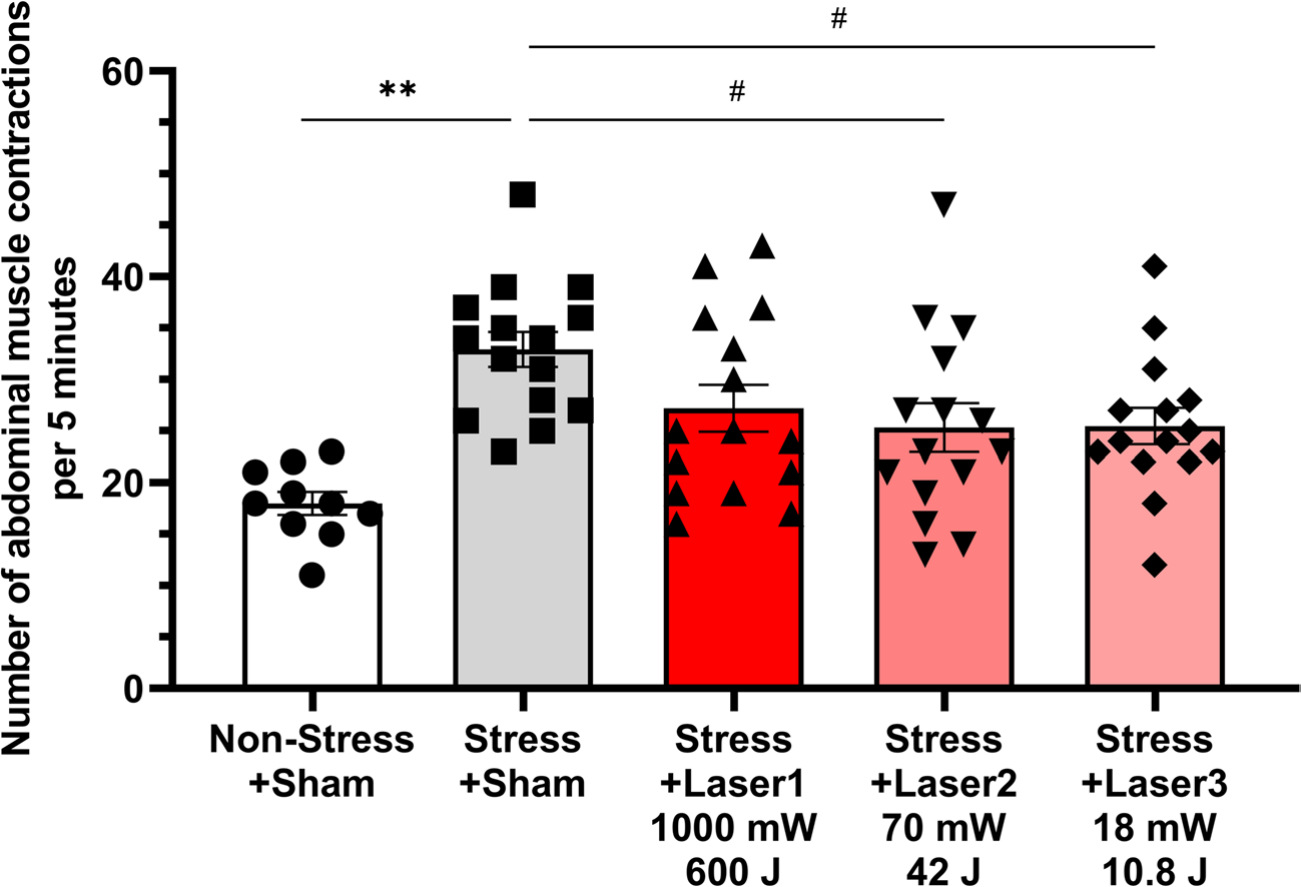
The effect of photobiomodulation on the number of abdominal muscle contractions during a 5-minute 60 mmHg balloon stimulation in the rectum (first test). The Stress + Sham group (number of abdominal muscle contractions: 32.9 ± 1.7) showed a significant increase in the number of abdominal muscle contractions compared to the Non-stress + sham group (18.0 ± 1.1). Although the Stress + Laser1 group (27.2 ± 2.3) exhibited a tendency to inhibit compared to the Stress + sham group, no statistically significant difference was observed. The Stress + Laser2 group (25.3 ± 2.4) and Stress + Laser3 group (25.5 ± 1.8) showed a significant decrease in the number of abdominal muscle contractions compared to the Stress + sham group. Data are presented as means ± SEM; either Dunnett’s multiple comparisons test or student’s t-test was used (*n* = 10 or 15); ** *p* < 0.01 using student’s t-test; # *p* < 0.05 using Dunnett’s multiple comparisons test vs. Stress + Sham group; SEM, standard error of the mean

The experimental procedures and a schematic diagram are presented in Fig. 1. Initially, an examination of three laser conditions was conducted (Fig. 2). The number of abdominal contractions in the Non-Stress + Sham group was 18.0 ± 1.1, whereas in the Stress + Sham group, it increased significantly to 32.9 ± 1.7 (*p* < 0.001). The number of abdominal contractions in the Stress + Laser1 group (average power 1000 mW, energy 600 J) was 27.2 ± 2.3, which did not differ significantly from the Stress + Sham group. In the Stress + Laser2 group (70 mW, 42 J), the number of abdominal contractions was 25.3 ± 2.4, which was significantly lower compared to the Stress + Sham group (*p* = 0.031). The Stress + Laser3 group (18 mW, 10.8 J) showed 25.5 ± 1.8 abdominal contractions, also indicating a significant decrease compared to the Stress + Sham group (*p* = 0.034).

**Fig. 3:**
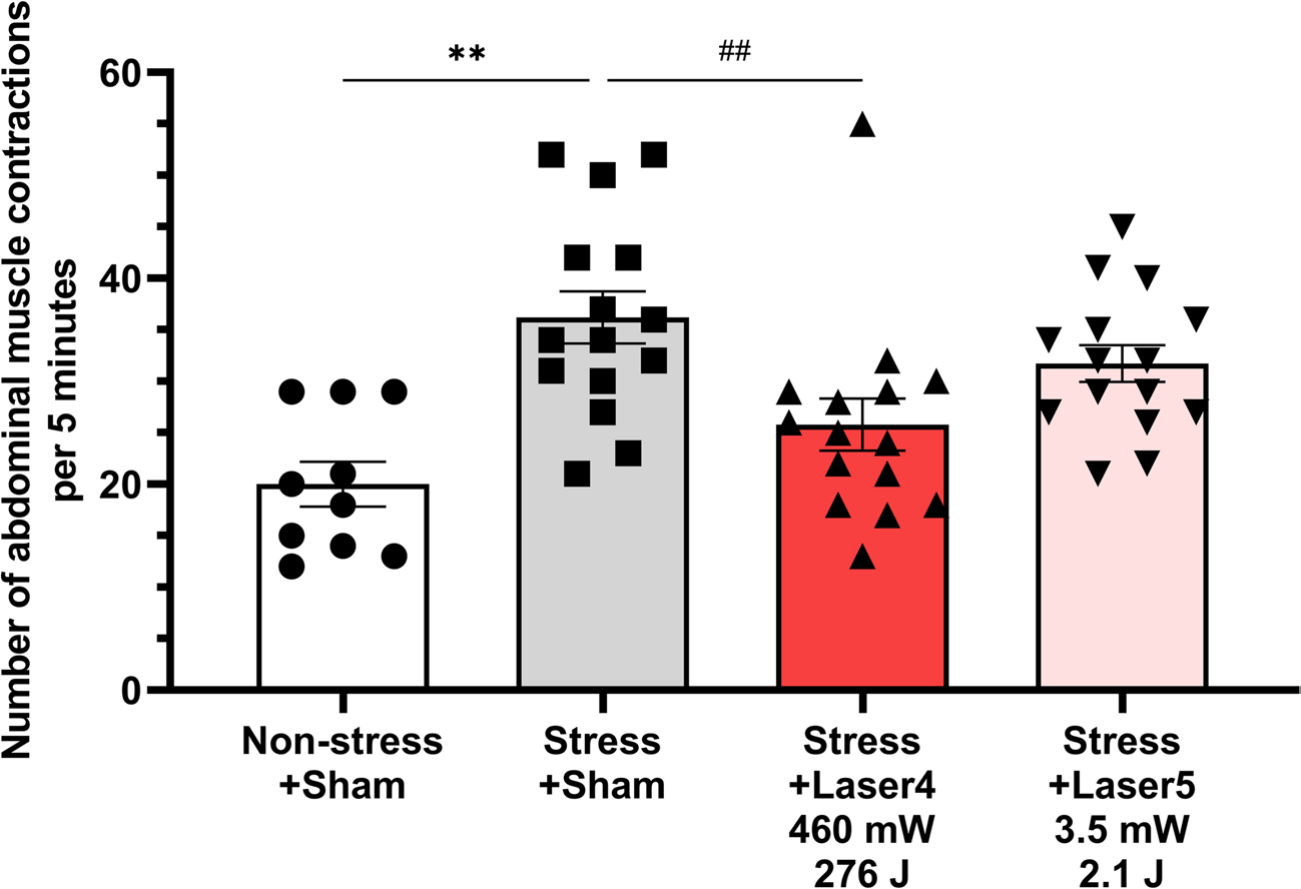
The effect of photobiomodulation on the number of abdominal muscle contractions during a 5-minute 60 mmHg balloon stimulation in the rectum (second test). The Stress + Sham group (number of abdominal muscle contractions: 36.2 ± 2.5) showed a significant increase in the number of abdominal muscle contractions compared to the Non-stress + sham group (20.0 ± 2.2), confirming the induction of the pathological condition. The Stress + Laser4 group (25.8 ± 2.5) exhibited a significant decrease in the number of abdominal muscle contractions compared to the Stress + sham group. No significant decrease in the number of abdominal muscle contractions was observed with the Stress + Laser5 group (31.7 ± 1.8) compared to the Stress + sham group. Data are presented as means ± SEM; either Dunnett’s multiple comparisons test or student’s t-test was used (*n* = 10 or 15); ** *p* < 0.01 using student’s t-test; ## *p* < 0.01 using Dunnett’s multiple comparisons test vs. Stress + Sham group; SEM, standard error of the mean

Subsequently, an examination of another two conditions was performed (Fig. 3). The number of abdominal contractions in the Non-Stress + Sham group was 20.0 ± 2.2, and in the Stress + Sham group, it increased significantly to 36.2 ± 2.5 (*p* < 0.001). The Stress + Laser4 group (460 mW, 276 J) had 25.8 ± 2.5 abdominal contractions, which was significantly lower compared to the Stress + Sham group (*p* = 0.005). The Stress + Laser5 group (3.5 mW, 2.1 J) showed 31.7 ± 1.8 abdominal contractions, which did not show a significant difference compared to the Stress + Sham group.

The results of immunohistochemical analysis indicated that the markers c-Fos, pERK1/2, mGluR5, and TRPV1 in the L6 spinal dorsal horn showed no significant differences between the Non-Stress + Sham group and the Stress + Sham group (data not shown).

## Discussion

This study demonstrated that percutaneous laser irradiation to the L6 dorsal root ganglion in a restraint stress model, one of the IBS models, suppresses the number of abdominal muscle contractions. These results suggest that lasers could potentially treat or prevent abdominal pain symptoms of IBS.

In this study, we employed the acute restraint stress model. This model, commonly used as an animal model for IBS, involves placing animals in a small cage to limit their movement, thereby exposing them to acute stress. This process can induce physiological and behavioral changes that mimic some symptoms of IBS [26]. The efficacy of treatments such as linaclotide [27] and ramosetron [28], which are clinically used for IBS, has been recognized using this model. Consequently, the effectiveness of lasers observed in the acute restraint stress model suggests the potential of lasers in treating the abdominal pain symptoms associated with IBS, as demonstrated in our research.

The laser was irradiated on the L6 dorsal root ganglion of the rats. The L6 dorsal root ganglion in rats is a pelvic visceral nerve, part of which projects to the rectum where the catheter was inserted [25]. The pelvic visceral nerves convey information from the colonic mucosa through Aδ and C fibers and transmit information about the colon to the central nervous system [23, 29]. The contraction of the abdominal muscles induced by inserting and inflating the catheter in the colon is known as the visceral motor response (VMR) [30], and there is a correlation between the severity of visceral hypersensitivity and the frequency of VMR [23, 31]. In VMR, Aδ and C fibers play a crucial role in transmitting nociceptive signals [23]. Electrophysiological studies have reported that PBM does not affect Aβ fibers but selectively inhibits Aδ and C fibers [19, 20]. Thus, it is suggested that the laser selectively inhibits Aδ and C fibers of the pelvic visceral nerves, potentially improving visceral hypersensitivity and reducing the frequency of abdominal contractions. Additionally, the pelvic visceral nerves in rats correspond to the sacral nerves in humans [32]. Therefore, in humans, PBM of the sacral nerves may have the potential to improve visceral hypersensitivity in IBS.

In the first experiment, conditions of 1000 mW, 600 J (Stress + Laser1), 70 mW, 42 J (Stress + Laser2), and 18 mW, 10.8 J (Stress + Laser3) were established. The average power of 1000 mW (10 W peak, 5 Hz, 10%Duty) setting was based on prior research where an 830 nm laser was percutaneously applied to the L6 dorsal root ganglion in a cystitis model rat and found to be effective [33]. Because some of the sensory nerves of both the bladder and colon project to the L6 dorsal root ganglion [32], and the laser was reported to inhibit the hyperactivity of sensory nerves in the cystitis model rat [33], this aligns with the hypothesized mechanism of action in our study. The 70 mW, 42 J and 18 mW, 10.8 J conditions were set to reproduce the laser intensity that would be reached by a 1000 mW, 600 J percutaneous irradiation of the human sacral foramen (approximately 20 mm depth from the skin surface [34]) at a depth of dorsal root ganglion of 11 mm in rats, based on our previous reports of penetration studies in rats [35]. In the second experiment, the 460 mW, 276 J (Stress + Laser4) and 3.5 mW, 2.1 J (Stress + Laser5) were set. 460 mW, 276 J was to verify whether a similar effect could be obtained with an output between 1000 mW, 600 J and 70 mW, 42 J, as the first trial indicated equivalent efficacy at 1000 mW, 600 J, 70 mW, 42 J, and 18 mW, 10.8 J. The 3.5 mW, 2.1 J condition was established to determine whether the effect would disappear at a lower power than 18 mW, 10.8 J.

These results hold significant implications for examining the relationship between laser efficacy and intensity. Particularly when considering the translation of these findings to clinical studies in humans, the intensity of a 1000 mW, 600 J laser at the nerve depth in rats, if replicated in the human sacral foramen, could pose a risk of thermal injury due to excessive laser strength and resultant temperature rise on the skin surface [36]. However, the effectiveness observed in rats at 70 mW, 42 J and 18 mW, 10.8 J suggests that in humans, treatment effects might be achievable under conditions with a relatively lower risk of thermal injury. This insight could provide an important guideline for balancing safety and efficacy in future applications to humans.

To elucidate the underlying mechanisms, we conducted immunostaining to assess potential markers such as c-Fos, pERK1/2, mGluR5, and TRPV1, in the L6 spinal dorsal horn. These markers did not differ between the Non-Stress + Sham group and the Stress + Sham group. This finding suggests that these markers are not suitable for evaluating the acute restraint stress-induced visceral hyperalgesia. Consequently, it may be inferred that the visceral hyperalgesia associated with abdominal muscle contractions does not correlate with alterations detectable by these markers.

Limitations of this study are discussed. First, due to issues with the experimental system, we could not evaluate the effect on bowel movement abnormalities, such as the frequency of defecation. Specifically, in the restraint stress model, the number of bowel movements during restraint stress is counted. However, because restraint during laser irradiation induces defecation, it was not possible to irradiate with the laser before restraint. Nevertheless, there are reports suggesting that diarrhea and constipation can occur due to abdominal pain [37, 38]. Therefore, it is possible that PBM could alleviate diarrhea and constipation by suppressing visceral pain hypersensitivity. Second, we conducted two separate trials, and the relationship between the 5 dose conditions in the same experiment has not yet been validated. Conducting studies with multiple appropriate dose groups in a single study will help validate the broad dose-response characteristics of PBM. Third, the restraint stress model used in this study is an acute model, whereas IBS is a chronic disease; the use of a chronic model, such as maternal separation [39], allows a detailed examination of whether PBM can be applied to IBS.

## Conclusion

We demonstrated that PBM improves visceral hyperalgesia in restraint stress model rats. This suggests that PBM could be a new treatment option for IBS patients.

## Data Availability

The original contributions presented in this study are included in the article, and further inquiries can be directed to the corresponding author.
